# Formation of Pus by Inflammatory Action

**Published:** 1868-01

**Authors:** 


### FORMATION OF PUS BY INFLAMMATORY ACTION.

In a remarkable discourse, most eloquently delivered before the Berlin Medical Society, Dr. Cohnheim detailed the results of his observations on the formation of pus as a product of inflammatory action. These results are of sufficient significance to mark a new era in the history of pathological science.

The generally accepted theory of Pyogenesis, which refers the origin of pus-corpuscles to the proliferation of cells or germinal matter in connective tissue, has received its death-blow.

The morphological resemblance of pus-corpuscles to white blood-cells has long been universally acknowledged. The modern discovery of the contractile properties with which they are both endowed, has tended still further to strengthen the belief in their intimate relationship. Dr. Cohnheim has now demonstrated their *identity* by proving that *pus-corpuscles are actually white cells which have emigrated from the blood-stream.*

He commenced his studies in the cornea, the classical ground for the study of inflammation. Availing himself of the well-known properties of white blood-cells to grasp and fix finely divided substances in their contractile stroma, he has been enabled to track these bodies, colored by aniline-blue injected into the blood, to the seat of inflammation, artificially excited in the cornea, and to recognize them as the cellular elements infiltrating the inflamed part. He has, moreover, succeeded, in a second series of observations, for which, for obvious reasons, a transparent vascularized tissue was selected, in actually observing step by step the emigration of the white corpuscles through the walls of the veins and capillaries of the inflamed mesentery into the surrounding tissues, and the pseudo-membranous fibrin effused on its surface.

The connection between these extraordinary facts and the well-known observations of Recklinghausen (Virchow's *Archiv*, 1863, vol. xxviii. pp. 157–197), on the presence of wandering contractile corpuscles in the plasmatic channels of the cornea, mesentery, and connective-tissue of other parts, will at once be evident.

Recklinghausen ventured upon no definite statement as to the origin of these bodies. He alluded to the probability of their being formed from the first connective-tissue corpuscles; but found it impossible to adduce any observation calculated to give support to this supposition. He had recognized their morphological identity with pus-cells, lymph, and white blood-corpuscles. He was acquainted with the increase and accumulation of these wandering elements, as "the essential change in the slighter degrees of inflammation;" but the chain of observations necessary to assign to them their true position and origin had to be completed by Dr. Cohnheim's elaborate investigations.

It is interesting to remark, for the purpose of illustrating the stages of continuity in scientific discovery that Recklinghausen had also demonstrated the possibility of contractile cells penetrating the corneal tissue from without by a very ingenious

ARMY MEDICAL LIBRARY WASHINGTON D. C. U. S. A.

experiment. He inserted pieces of cornea and finely powdered vermilion into the lymph-sacks of living frogs, and found them on removal after a certain time infiltrated with wandering lymph-corpuscles laden with granules of vermilion.—*Brit. Med. Jour.*, June 22, 1867.—*Med. News.*

